# Working to Increase Stability through Exercise (WISE): screening, recruitment, and baseline characteristics

**DOI:** 10.1186/s13063-021-05761-0

**Published:** 2021-11-15

**Authors:** Christopher N. Sciamanna, Noel H. Ballentine, Melissa Bopp, Vernon M. Chinchilli, Joseph T. Ciccolo, Gabrielle Delauter, Abigail Fisher, Edward J. Fox, Suzanne M. Jan De Beur, Kalen Kearcher, Jennifer L. Kraschnewski, Erik Lehman, Kathleen M. McTigue, Edward McAuley, Anuradha Paranjape, Sol Rodriguez-Colon, Liza S. Rovniak, Kayla Rutt, Joshua M. Smyth, Kerry J. Stewart, Heather L. Stuckey, Annie Tsay

**Affiliations:** 1 Department of Medicine, Division of General Internal Medicine, Penn State College of Medicine, Box HO34, 500 University Drive, Hershey, PA 17033 USA; 2 Penn State College of Medicine, Hershey, USA; 3 Penn State University, State College, USA; 4 Columbia University, New York City, USA; 5 Johns Hopkins University, Baltimore, USA; 6 University of Pittsburgh, Pittsburgh, USA; 7 University of Illinois, Urbana, USA; 8 Temple University, Philadelphia, USA

**Keywords:** Falls, Physical activity, Osteoporosis, Fall-related, Exercise

## Abstract

**Background:**

The aim of this paper is to describe the utility of various recruitment modalities utilized in the Working to Increase Stability through Exercise (WISE) study. WISE is a pragmatic randomized trial that is testing the impact of a 3-year, multicomponent (strength, balance, aerobic) physical activity program led by trained volunteers or delivered via DVD on the rate of serious fall-related injuries among adults 65 and older with a past history of fragility fractures (e.g., vertebral, fall-related). The modified goal was to recruit 1130 participants over 2 years in three regions of Pennsylvania.

**Methods:**

The at-risk population was identified primarily using letters mailed to patients of three health systems and those over 65 in each region, as well as using provider alerts in the health record, proactive recruitment phone calls, radio advertisements, and presentations at community meetings.

**Results:**

Over 24 months of recruitment, 209,301 recruitment letters were mailed, resulting in 6818 telephone interviews. The two most productive recruitment methods were letters (72% of randomized participants) and the research registries at the University of Pittsburgh (11%). An average of 211 letters were required to be mailed for each participant enrolled. Of those interviewed, 2854 were ineligible, 2,825 declined to enroll and 1139 were enrolled and randomized. Most participants were female (84.4%), under age 75 (64.2%), and 50% took an osteoporosis medication. Not having a prior fragility fracture was the most common reason for not being eligible (87.5%). The most common reason provided for declining enrollment was not feeling healthy enough to participate (12.6%).

**Conclusions:**

The WISE study achieved its overall recruitment goal. Bulk mailing was the most productive method for recruiting community-dwelling older adults at risk of serious fall-related injury into this long-term physical activity intervention trial, and electronic registries are important sources and should be considered.

## Background

Injuries from falls are a leading cause of death among older adults. Between 2000 and 2013, the age-adjusted fall injury death rate among adults over 65 increased from 29.6 per 100,000 to 56.7 per 100,000 [1]. Given these trends, along with the projected growth in the number of adults over 65 in the USA; from 43 million in 2012 to 73 million by 2030 [2], there is an urgent need to develop and test interventions to reduce falls from injuries among older adults.

We recently completed recruitment for an randomized controlled trial of an exercise intervention designed to reduce falls and fractures. The study, called *Working to Increase Stability through Exercise* (WISE), recruited adults over age 65 who had suffered a fragility fracture in the past 10 years (e.g., vertebral fracture or fall-related). Participants were randomized to either an enhanced usual care condition, which included health information and ordering a clinical bone density scan, or to an intervention condition. The intervention is a 3-h per week strength and balance training program offered for 36 months. Participants in the intervention group are given the option of joining in-person, in any of 25 community-based exercise sites (e.g., churches, community centers) established across the 3 sites, or at home by DVD. The primary outcome measure is the rate of injuries that lead to the need for medical care, reported by phone every 4 months and verified with a medical record review, which we refer to as serious fall-related injuries (SFRI). We have described the methods for the study elsewhere [3].

Recruitment is often the most challenging part of a clinical trial. Up to 95% of trials experience delays due to challenges in recruitment [4] and up to 19% of trials are closed due to low rates of recruitment [5]. A 2018 systematic review examined strategies to enhance enrollment and identified only three that were supported by strong evidence: (1) open trials rather than blinded, placebo trials; (2) telephone reminders to people who do not respond to a mailed invitation, and (3) user testing to optimize recruitment leaflets [5]. More recently, a systematic review compared online (e.g., Facebook, Google search) versus offline recruitment (e.g., mailings) and observed that online recruitment led to a significantly greater rate of recruitment at a significantly lower cost [6]. As internet access rates among older adults are lower than for younger adults, internet recruitment sources for older adults are often not considered. As of 2021, however, 75% of older adults have internet access, with access rates continually rising [7] and 50% of older adults use Facebook [8], making online recruitment increasingly viable for older adults.

A significant challenge in longer-term lifestyle studies (e.g., diet, exercise) is recruiting enough individuals who reflect the target population and are willing to participate in a multi-year intervention trial. Successful strategies to recruit aging, medically at-risk adults are especially needed as supervised physical activity interventions have demonstrated an average effect size of Cohen’s *d*=0.62 in improving physical function measures [9], yet fewer than 1 in 20 older adults meet activity guidelines [10]. In this report, we describe the successes and challenges of completing recruitment across three cities, as well as the yields from different recruitment methods and a baseline examination of the key characteristics of our study population.

## Methods

### Institutional Review Board (IRB) approval

The study was approved by the Institutional Review Boards of the Penn State College of Medicine, the University of Pittsburgh, and the Lewis Katz School of Medicine at Temple University, which we will refer to as Penn State, Pittsburgh and Temple, respectively. Each IRB approved each protocol element separately, in an arrangement that required Penn State’s IRB to first approve protocol elements before being evaluated by the other institution’s IRB. If any of the protocol changes were rejected by the IRB at any of the three institutions, the protocol was updated at all three sites to ensure consistency. A more detailed description of the methods is included in the protocol manuscript [3].

### Community Advisory Board

In order to optimize the patient-centeredness of all aspects of the project, including recruitment, we formed a Community Advisory Board (CAB) at the initiation of the project. We engaged leaders and stakeholders from health insurance companies (e.g., Highmark, Aetna), older adult services organizations (e.g., Area Agency on Aging), governmental agencies (e.g., Pennsylvania Department of Health), and volunteer groups (e.g., Retired & Senior Volunteer Program), as well as peer leaders and participants from the 15 original exercise sites that served as pilot locations for the trial. The CAB met in person in the first year of the project and then every 1–3 months by phone during the recruitment phase. The CAB’s role was to provide advice and feedback about study methods, potential partnerships to optimize recruitment and to review recruitment materials.

### Staff training

Prior to starting recruitment, a training session was held for the project managers at each recruitment site. This session included a review of the standard operating procedure (SOP) documents as well as a review of materials needed to conduct the baseline visits. After this initial training, the project managers at each recruitment site were responsible for training staff. Trainings for research staff involved a full review of the study protocol and SOPs for each work procedure. These SOPs were created by project managers at Penn State and were kept current in an online repository accessible to research staff at all three sites. For quality control purposes, quarterly in-person site visits were conducted by the Core project manager at Penn State to the University of Pittsburgh and Temple sites. During these visits, the Core project manager audited the recruitment and enrollment processes. In-person, site-specific staff training was then tailored and conducted based upon the audit findings. Additional in-person staff training was conducted during the Annual Stakeholder Meeting.

### Eligibility criteria

The eligibility criteria were designed to limit exclusions, in keeping with pragmatic trial design [11]. Eligibility criteria included: age ≥ 65, history of a fragility fracture in the past decade, able to walk 100 feet, a negative screening test for dementia, and permission from a primary care provider. We initially focused on those who suffered a FF in the past five years, but since we could not yield enough individuals to meet study enrollment goals, the window for FF was expanded to 10 years given that the risk of secondary FF persists. Where possible, contacts were made with individuals with the highest likelihood of being 65 or older and having a FF in the past decade. For example, letters were sent to individuals with a history of osteoporosis in order to increase the likelihood of identifying individuals with a fragility fracture, though the eligibility criteria remained consistent in that individuals needed to have a history of a fragility fracture to be enrolled.

### Baseline visit

Prior to the baseline visit, a consent form was mailed to participants to review before the in-person visit. During the baseline in-person visit, the study was explained in detail, and randomization was performed, stratified by site (Pittsburgh, Hershey-Harrisburg, Philadelphia), gender, and prior or current use of a potent medication for osteoporosis (e.g., alendronate). Self-reported survey measures were collected (e.g., falls history, loneliness) and several directly observed measures were taken (e.g., blood pressure, chair stand performance).

### Enrollment: goal

The original goal was to recruit and randomize 2000 individuals, based on a power analysis to detect a 3% difference (7% v. 4%, a 43% relative risk reduction) in new fragility fractures, the original primary clinical outcome. Due to rates of enrollment being lower than anticipated, the enrollment goal was modified to 1130 individuals, and the primary clinical outcome was changed to a 6% difference (13% v. 7%, a 46% relative risk reduction) in the composite outcome measure of fragility fracture and serious fall-related injury (FF/SFRI). The original proposal included power and sample size analyses for both clinically relevant outcomes and the original sample size left the study overpowered to detect a difference in FF/SFRI, with power estimates ranging from 94–99% under a range of assumptions of the rate of FF/SFRI and treatment effect. This change was consistent with the overall aim of the study, to test the impact of an exercise program on a clinical outcome which, if positive, may provide a rationale for an exercise program to be included as a standard Medicare benefit for older adults at risk of a fall-related injury [12, 13]. The study had a racial/ethnic minority enrollment goal of 18%, slightly lower than the statewide rate of 21%, based on 2012 Census data, given the lower incidence of osteoporosis in Black, compared to White, women [14].

### Enrollment: methods

A variety of recruitment methods were used, in order to ensure timely enrollment. Each site sent recruitment letters and, where possible, those letters were targeted to individuals who were likely to meet the inclusion criteria. Patients of each site had been informed, in their respective Notice of Privacy Practices for Protected Health Information, that they may be contacted for a future research study. Letters were targeted to three groups of people age 65 or older: (1) those who had a previous fragility fracture, (2) those with a history of osteoporosis, and (3) those without either medical condition in their history. Two main sources were employed to identify eligible individuals: each institution’s electronic medical record (EMR), which included fragility fracture and osteoporosis history, and marketing lists purchased from Lorton Data (www.lortondata.com), which allowed us to target individuals with a self-reported osteoporosis history. Patients of each site are informed, in their respective Notice of Privacy Practices for Protected Health Information, that they may be contacted for a future research study. For EMR queries, International Classification of Disease (ICD) codes for fragility fracture (FF) (e.g., 812.0 - Fracture of Upper End of Humerus, closed) were used to identify individuals who had likely suffered a FF in the past 10 years, making them eligible for the current study. This code list was derived from prior quality improvement work by one of the co-investigators (EF) [15]. As ICD 10 codes were implemented in 2015, both ICD 9 and ICD 10 codes were used to identify eligible participants.

In addition, several site-specific recruitment options were employed. The University of Pittsburgh, for example, used two research registries to identify potential participants. The Pepper Registry (www.pepper.pitt.edu) included the names of older adults who had previously expressed an interest in future studies specific to older adults. The Pitt+Me registry (www.pittplusme.org), overseen by the University of Pittsburgh’s Clinical and Translational Science Institute (CTSI), allows individuals to join and be notified by the staff of future studies that are relevant to their conditions or interests. The University of Pittsburgh also identified participants by using an electronic alert embedded in the EMR. During clinical encounters, this alert flagged patients that met key inclusion criteria so that the provider could consider referring the patient to the study. In several instances, recruitment methods were pilot tested at one site before being implemented at the other two sites. For example, letters allowing individuals to opt-out of being called about the study were pilot tested at Penn State, but the results suggested that the method was not successful enough to be used across all sites. Traditional marketing methods were also used across sites to recruit participants, including radio advertisements, on-hold audio messages, and presentations at community meetings (i.e., 50+ Festivals).

### Enrollment: phone interviews

Screening was conducted primarily by phone. In some cases, such as at community events, screening questions were completed in person. Potential participants were screened for eligibility and study expectations (i.e., study visits timing, randomization) were discussed. To save time, the sequence of topics presented on the phone was modified over the first 6 months to move the issues most likely to lead to ineligibility (e.g., age < 65) or disinterest (e.g., time commitment, travel burden) to the beginning of the screening process. For example the intervention was expected to require 150 h of time over 3 years and in-person attendance was encouraged, though transportation was not provided, which was recognized early in the recruitment process as a significant barrier to participation. During phone interviews, the name of the participant’s primary care provider (PCP) was asked and a dementia screener was performed [16]. If the dementia screen was positive, a set of questions were then asked to assess the participant’s understanding of the nature of the study. If any answers were incorrect, the participant was considered ineligible. After the phone interview, PCPs were contacted by fax to medically clear the participant for the exercise portion of the intervention, and to order a dual-energy X-ray absorptiometry (DXA) scan to determine the bone mineral density (if not already performed in the previous 2 years and medically indicated). Given the pragmatic nature of the study design, the DXA test ordering was performed based on the provider’s understanding of the clinical appropriateness of ordering the test. If a DXA had been performed within the past 2 years, for example, we instead planned to obtain the prior result via chart review at the end of the study. As a result, the baseline DXA characteristics are not presented here.

### Enrollment: strategies to enhance enrollment

To better track the recruitment process, we used Tableau (www.tableau.com) to visualize our progress and to automate weekly reports that, based on recruitment trends, estimated the date when enrollment would be completed. Despite lowering the enrollment goal for the study (as above) to 1130, the rate of enrollment continued to be a challenge. Therefore, after several discussions with our Community Advisory Board (CAB) to seek ideas, we undertook several specific tactics to enhance recruitment. For each of the following tactics, the CAB was integral to reviewing materials and suggesting content and edits.

First, we increased our marketing for the study in several ways. We began advertising at events for vendors of products and services for older adults, across the three regions, by paying for a table at the event, making posters, and sending a staff member to speak to people who expressed interest. We also created a video about the study and placed it on a study website (www.wisestudy.org) and the URL was placed on the recruitment letter. We also worked with our marketing departments to identify media outlets that may be interested in doing a story about the study. As a result, the Philadelphia Inquirer and the National Public Radio affiliate in Central Pennsylvania (www.witf.org) both reported on the study. Links to both stories were then placed on our study website.

Second, in 2017, with the CAB’s assistance, we created a new version of the recruitment letter that included specific changes that had previously been proven to enhance behavior changes [17, 18]. First, the letter included the number of participants that had been recruited to date, to give a sense of “social proof” as well as a deadline for recruitment, to give a sense of scarcity, both of which are tactics proven to change behavior. Second, the letter included a link to a website that included a video, featuring study participants in both conditions explaining the importance of the study and giving testimonials about their experience. Third, the letter mentioned the stories in the local news (as above), in order to convey a sense of expertise or authority. We then tested the revised letter against the former letter by mailing letters to a random assignment of addresses, to understand whether the revised letter led to a greater number of participants being enrolled than the original letter.

Third, in 2017, we began making proactive outbound recruitment phone calls. The recruitment letter was changed to note that study staff may call by phone, unless the individual called the included toll-free number or mailed back a letter that was included, noting that the individual did not want to be called. This proactive outreach process has been shown to be more effective at enhancing enrollment in clinical trials than the reactive processes that we had previously been using [19].

Fourth, in 2017, it became clear that the project was not meeting its minority recruitment goal of 18%, so a number of actions were taken to enhance minority recruitment. These changes were implemented mainly at the University of Pittsburgh and Penn State sites, as their minority enrollment rates, defined as the percent of Black or African American plus Hispanic or Latino participants, were the lowest. At Penn State, enrollment was expanded to two local cities, York and Lancaster, which had a higher percentage of minorities (63.8% and 38.9%, respectively) than the state overall (24.3%). At the University of Pittsburgh, the following activities were initiated: (1) presentations about the study were made at community health centers in minority neighborhoods (e.g., Homewood Community Engagement Center, Kingsley Center); (2) mailings were prioritized to neighborhoods with the highest percentage of minorities, so long as the planned exercise sites in those communities were in neighborhoods with a sufficient population of older adults and were safely accessible to older adults; and (3) advertisements were placed in media outlets with a higher percentage of minority readers (e.g., Soul Pitt Magazine)

Fifth, in the latter half of 2018, we greatly increased the number of letters being mailed, particularly at the Penn State site where we did not have access to the registries and electronic health record reminders available at the University of Pittsburgh.

### Measures

Baseline demographics were obtained by self-report using questions from the National Health Interview Survey (NHIS) that measured age, gender, ethnicity, race, smoking status, and past medical history of hypertension and osteoporosis [20]. Baseline history of falls was obtained by self-report using a question from the Behavioral Risk Factor Surveillance System (BRFSS) about the number of falls in the past year [21]. Emergency room visits and hospital admission in the past year were assessed via self-report using modified questions from Project RED (Re-Engineered Discharge) [22]. Medication use for osteoporosis was assessed by asking whether participants currently or previously took any of 10 specific prescription medications for osteoporosis (e.g., alendronate, denosumab, teriparatide). Height and weight were measured (for BMI calculation) using a wall-mounted stadiometer and validated scale [23]. Blood pressure and heart rate were measured using the average of three readings from a validated automated machine (Omron HEM 907XL) [24]. Physical performance was assessed by measuring the maximum number of chair stands and arm curls (5-pound dumbbells for women and 8-pound dumbbells for men) that could be performed properly in 30 s [25]. Symptoms of fatigue, pain, depression, and anxiety were assessed using scales from the Patient Reported Outcomes Measurement Information System (PROMIS) [26, 27]. Loneliness was assessed using the 3-item Brief Loneliness Questionnaire [28].

### Statistical analysis

Descriptive statistics were used to characterize study participants and enrollment rates (i.e., number of participants enrolled per week) as well as the impact of different recruitment strategies. Chi-square tests for categorical variables and Student’s *t* tests, analysis of variance, Wilcoxon rank sum tests, or Kruskal-Wallis tests for continuous variables were used to compare participants in each study condition as well as at each study location.

## Results

**Fig. 1:**
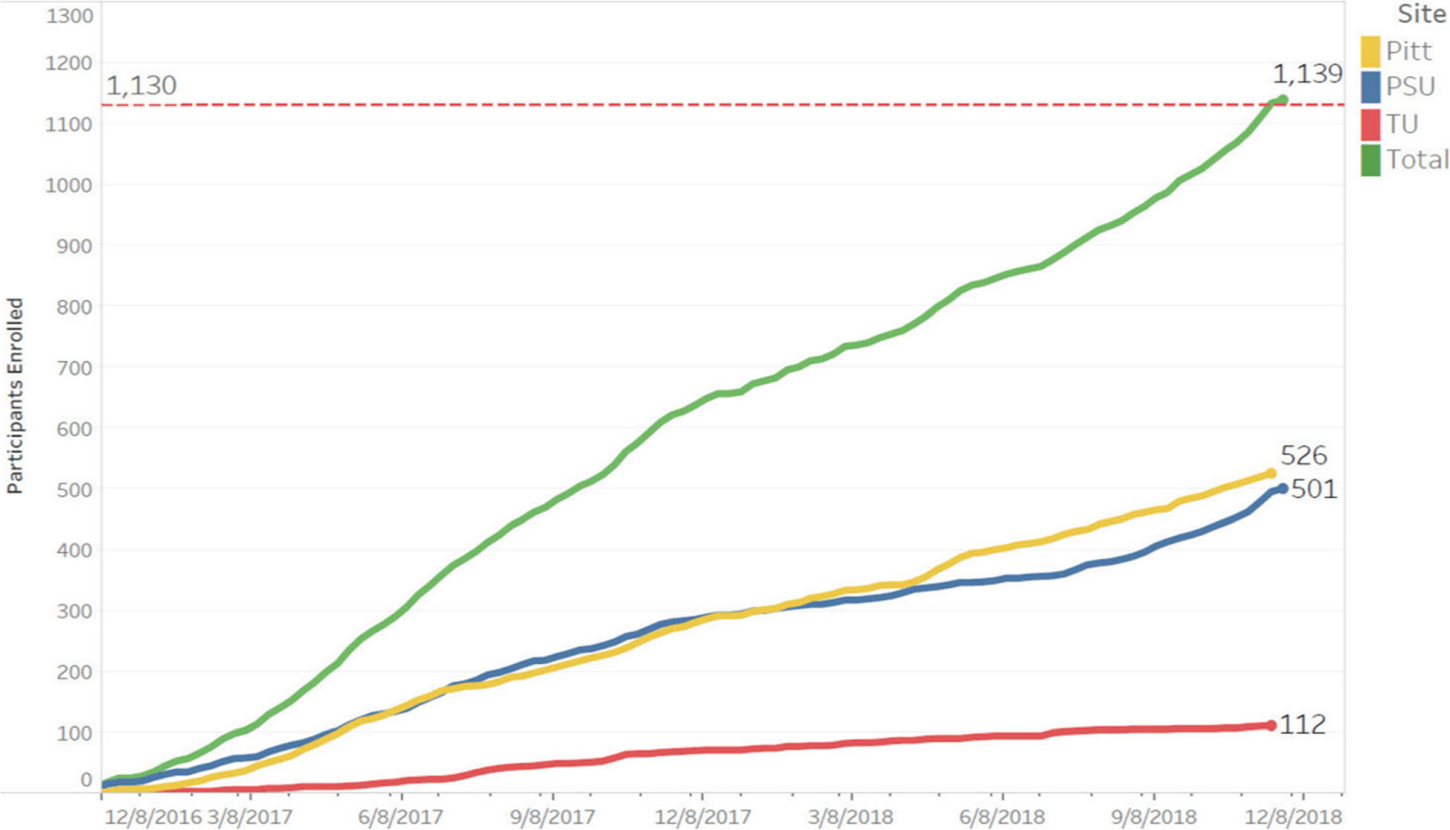
Enrolled participants over time

Enrollment began in December 2016 and ended in November 2018 with 1139 participants being enrolled, for a rate of approximately 13 participants per week over 2 years (see Fig. 1). In 2017 the two letters were tested against each other, with 11,022 letters being randomly assigned to different addresses. While 0.9% of the newer letters led to a screening (versus 0.6% of the original letters), the enrollment rates for both were equal to 0.1%. In 2017, 1945 proactive recruitment phone calls were made, resulting in 1162 interviews and 41 subjects (3.5% of interviews) being enrolled. In the second half of 2018, the Penn State site increased the number of letters mailed each week, from 623 per week to 3627 per week (a 5.8 times increase), leading to increases in average incoming calls (12 versus 66; a 5.5 times increase) and increase in enrollment (2 versus 6; a 3.0 times increase).

**Fig. 2:**
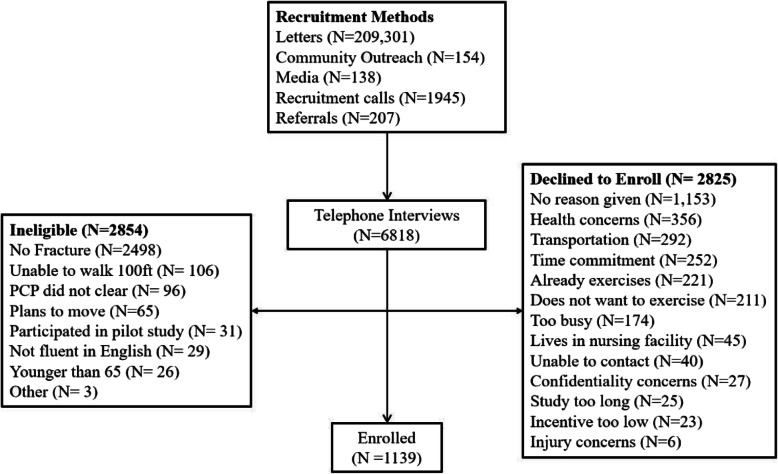
WISE recruitment funnel

The recruitment flow is shown in Fig. 2. In total 209,301 letters were mailed, 6818 telephone interviews were conducted, and 1139 were ultimately randomized, slightly above the goal of 1130. We conducted 6.0 interviews for every participant enrolled. For the 2854 individuals who were ineligible, the main reasons were not having a fragility fracture in the past 10 years (2498; 87%) and reporting inability to walk 100 feet (106; 3.7%). For the 2825 individuals that declined to enroll, most gave no specific reason (1153; 40.8%) and the main reasons provided were not feeling healthy enough to participate (356; 12.6%), concerns over transportation to the exercise sessions (292, 10.3%), and concerns about the long time commitment (252; 8.9%).

Table 1 shows the source of recruited participants. Most (72%) participants were recruited by screening letters, with small percentages coming from a range of other sources, including research registries available at the University of Pittsburgh (11%), referral from physicians (6%), and others. As a result of having additional recruitment methods available, the University of Pittsburgh enrolled only 57% of participants using mailings, versus 87% and 86% for Penn State and Temple, respectively. In addition, the University of Pittsburgh had an electronic health record alert which was activated, so they were able to recruit 10% of their participants (52 of 526) via referral, compared to only 2% at both Penn State and Temple.

**Table 1:** WISE recruitment by source and site

	Penn State		Pitt		Temple		Total	
	#	%	#	%	#	%	#	%
Recruitment letters	436	87	300	57	86	77	822	72
Research registry	n/a	n/a	126	24	n/a	n/a	126	11
Referral	9	2	52	10	2	2	63	6
Recruitment calls	26	5	14	2	1	1	41	4
Flyers/newsletters	3	1	20	4	7	6	30	3
Media (e.g. radio ad)	13	2	1	< 1	11	10	24	2
Community outreach	9	2	9	2	-	-	18	1
Other	5	1	4	< 1	5	4	15	1
Total	501	100	526	100	112	100	1139	100

Table 2 shows the yield by letter type, which is limited to the Penn State site, which instituted a more detailed letter tracking system, outside of REDCap, than the other sites. Overall, Penn State mailed 211 letters for every participant enrolled, though the number of letters mailed for each participant enrolled varied by a factor of 10 between different sources of participants. For example, the best yield was for letters to patients identified in the electronic health record (EHR) as having had a fragility fracture in the past (65 letters mailed per participant enrolled). The poorest yield was from a list purchased from a marketing company (Lorton Data), which required 847 letters to be mailed for each participant enrolled. Overall, response rates were higher using letters mailed to individuals who had been patients at Penn State Health, regardless of the risk factor being used (e.g., osteoporosis), than were response rates to letters mailed to individuals from the purchased list.

**Table 2:** WISE recruitment yield by electronic health record (EHR) and purchase list mailing type at Penn State

	Letters mailed	# Enrolled	Yield per 100 letters	Letters mailed per participant
Osteoporosis from purchased list	15,298	67	0.44	228
Age 65+ from purchased list	44,870	53	0.12	847
Fragility fracture from EHR	12,473	191	1.53	65
Osteoporosis from EHR	14,855	135	0.91	110
Fall History from EHR	10,178	17	0.17	599
Total	97,674	463	0.47	211

Table 3 shows the results of the telephone interviews across the study and by study site. Overall, 42% of individuals interviewed were ineligible, 41% declined to participate and 17% were enrolled. Ineligibility was highest at Penn State (46%) and lowest at the University of Pittsburgh (39%). The highest rate of individuals declining to enroll was at the University of Pittsburgh and Temple (46% at both sites) and the lowest rate at Penn State (33%). Enrollment, as a percentage of telephone interviews, was highest at Penn State (21%) and lowest at Temple (13%). Across the study, 5.98 telephone interviews were conducted per participant enrolled.

**Table 3:** WISE recruitment yield of telephone interviews by site

	Telephone interviews	Ineligible		Declined participation		Enrolled	
		#	%	#	%	#	%
Penn State	2405	1110	46	794	33	501	21
Pitt	3565	1398	39	1641	46	526	15
Temple	848	346	41	390	46	112	13
Total	6818	2854	42	2825	41	1139	17

Baseline characteristics of participants at all sites are listed in Table 4. Across the study, most participants were under 75 years of age (64.3%), female (84.4%), and college-educated (54.9%). Minority recruitment was only 8.6%, less than half of the original goal of 18%. Half of the participants had used a medication for osteoporosis (e.g., alendronate) and more than half (50.9%) reported falling in the past year. Control and intervention conditions differed according to several baseline variables, including smoking status, recent hospitalization, and the number of bicep curls that could be done in 30 seconds. The remaining demographic, medical history, health care utilization, and physical examination variables were similar between conditions.

**Table 4:** WISE baseline data by condition

Characteristic (percent or mean)	Overall (***N***=1139)	Control (***N***=569)	Intervention (***N***=570)	***P*** value*
Age ≥ 75	35.8	35.2	36.4	0.665
Female	84.4	84.2	84.6	0.860
White, non-Hispanic	91.4	92.5	90.3	0.203
BMI	28.6 ± 6.5	28.6 ± 6.5	28.6 ± 6.5	0.995
Blood pressure, systolic	129.5 ± 17.7	129.7 ± 17.7	129.4 ± 17.8	0.750
Blood pressure, diastolic	73.3 ± 11.4	72.9 ± 11.7	73.8 ± 11.1	0.188
Heart rate	69.9 ± 10.8	70.3 ± 11.1	69.6 ± 10.4	0.300
College graduate	54.9	52.2	57.6	0.068
Osteoporosis medication, ever	50.0	49.9	50.0	0.976
Nonsmoker	97.0	98.2	95.7	**0.015**
History, hypertension	51.1	52.1	50.3	0.548
History, osteoporosis	51.2	51.0	51.3	0.909
Fall, past year	50.9	49.1	52.7	0.228
Lack companionship, hardly ever	69.8	69.3	70.3	0.719
Bicep curls, 30 s	12.4 ± 4.4	12.0 ± 4.3	12.7 ± 4.6	**0.012**
Chair stands, 30 s	9.0 ± 4.0	8.9 ± 4.0	9.0 ± 4.1	0.624
PROMIS, depression (20 = worst)	5.2 ± 2.3	5.3 ± 2.3	5.1 ± 2.4	0.179
PROMIS, anxiety (20 = worst)	5.8 ± 2.4	5.8 ± 2.4	5.8 ± 2.4	0.774
PROMIS, pain intensity (10 = worst)	2.8 ± 2.3	2.9 ± 2.4	2.7 ± 2.3	0.452
PROMIS, fatigue (20 = worst)	7.8 ± 3.3	7.9 ± 3.2	7.7 ± 3.3	0.290
ER visit in past year, yes	37.3	36.4	38.2	0.528
Hospital stay in past year, yes	21.5	18.4	24.7	**0.012**

*Percent with chi-square test; mean ± SD with two-sample *t* test or Wilcoxon rank sum test

Table 5 shows the baseline data by site. The percentage of minority participants was higher at Temple (42.3%) compared to either Pittsburgh (6.9%) or Penn State (2.7%). Osteoporosis history and use of osteoporosis medications across sites was similar, though the percentage of participants who fell in the past year was significantly different (*p*=0.039) at Penn State (54.5%), Pittsburgh (46.6%), and Temple (54.5%). Self-reported health, measured using Patient-Reported Outcomes Measurement Information System (PROMIS) instruments for fatigue, depression, anxiety, and pain intensity, differed significantly between sites, with Temple having poorer self-reported health for each of the above measures. Of note, a protocol deviation was identified in the way that chair stands were measured at Temple, so this variable is not included in the analysis at this site.

**Table 5:** WISE baseline data by site

Characteristic (percent or mean)	Overall (***N***=1139)	Penn State (***N***=501)	Pitt (***N***=526)	Temple (***N***=112)	***P*** value*
Age ≥ 75	35.8	34.9	36.0	39.1	0.708
Female	84.4	82.6	85.7	85.7	0.359
White, non-Hispanic	91.4	97.3	93.1	57.7	**< 0.001**
BMI	28.6 ± 6.5	29.0 ± 6.8	28.3 ± 6.3	28.3 ± 6.0	0.226
Blood pressure, systolic	129.5 ± 17.7	130.2 ± 17.3	127.5 ± 17.0	136.4 ± 20.7	**< 0.001**
Blood pressure, diastolic	73.3 ± 11.4	72.4 ± 11.7	74.2 ± 10.7	72.9 ± 13.1	**0.046**
Heart rate	70.0 ± 10.8	69.3 ± 11.2	70.1 ± 10.1	71.9 ± 12.2	0.066
College graduate	54.9	48.7	62.9	44.1	**< 0.001**
Osteoporosis medication, ever	50.0	50.1	49.8	50.0	0.996
Nonsmoker	97.0	97.1	97.0	96.4	0.931
History, hypertension	51.2	54.5	45.9	60.7	**0.003**
History, osteoporosis	51.2	53.5	48.7	52.8	0.317
Fall, past year	50.9	54.5	46.6	54.5	**0.039**
Lack companionship, hardly ever	69.8	67.4	73.1	64.3	0.059
Bicep curls, 30 s	12.4 ± 4.4	12.3 ± 4.7	11.6 ± 3.6	16.0 ± 5.1	**< 0.001**
Chair stands, 30 s	9.0 ± 4.0	8.5 ± 4.4	9.4 ± 3.7	N/A	**< 0.001**
PROMIS, depression (20 = worst)	5.2 ± 2.3	5.5 ± 2.7	4.9 ± 1.9	5.3 ± 2.5	**0.009**
PROMIS, anxiety (20 = worst)	5.8 ± 2.4	6.0 ± 2.6	5.5 ± 2.1	6.3 ± 2.7	**0.005**
PROMIS, pain intensity (10 = worst)	2.8 ± 2.3	2.8 ± 2.2	2.6 ± 2.3	3.6 ± 2.9	**0.004**
PROMIS, fatigue (20 = worst)	7.8 ± 3.3	8.2 ± 3.4	7.4 ± 3.1	8.2 ± 3.3	**<0.001**
ER visit in past year, yes	37.3	38.0	34.9	44.6	0.143
Hospital stay in past year, yes	21.5	21.3	21.1	24.3	0.751

*Percent with chi-square test, mean ± SD with ANOVA or Kruskal-Wallis test

## Discussion

Over 24 months of recruitment, the WISE study was able to meet its revised goal by enrolling 1139 older adults with a previous history of fragility fracture. A number of efforts were made to enhance enrollment, including testing a new version of the recruitment letter, using outbound, proactive recruitment phone calls, and increasing the number of letters mailed, though most of these efforts were unsuccessful in increasing the rate of enrollment. Despite efforts to modify the recruitment letter, for example, by utilizing proven tactics of communication and in partnership with the CAB, the revised letter was no more effective than the original letter. The reasons for this are not clear though it is quite possible that some of the changes made increased the impact of the letter while other changes decreased the impact of the letter. A more effective strategy for modifying the recruitment letter may have been to test each change individually, by using a digital platform such as Facebook. While only 73% of adults over 65 had access to the Internet in 2019, 46% use Facebook [29]. Facebook has built-in features that allow for rapidly testing text and graphical elements to identify the highest performing combination [30]. Also, as the proactive recruitment phone calls required 28 interviews to enroll one subject, compared to six for the study overall, they were deemed too labor-intensive to scale up. In the end, given the importance of mailed letters to our overall enrollment plan, greatly expanding the number of letters mailed was instrumental in allowing us to meet our recruitment goal and could have been increased far earlier.

While an array of recruitment methods were used, the majority of participants (73%) were recruited using letters, similar to other large-scale physical activity studies among older adults. The LIFE study, for example, enrolled 57% of 1635 participants using mailed letters [31], and the STRIDE study recruited 100% of 5451 participants using mailed materials [32]. Our method differed from the STRIDE study, a primary prevention trial of fall-related injuries, in that STRIDE mailed study information and asked individuals to return a brief screening postcard, containing three screening questions. While these methods are hard to compare, as they were functionally different, the percentage of letters that led to a phone screener in STRIDE (8.2%) was more than twice as the percentage of letters that led to a telephone interview in WISE (3.2%). This difference may be because the STRIDE mailed materials only to patients that had an existing relationship with one of the 86 enrolled practices. Similarly, in WISE, we observed that it required more than twice as many recruitment letters to enroll a participant using a commercial mailing of individuals with osteoporosis than when using a list of patients with osteoporosis who had an existing relationship with Penn State (228 v. 110, Table 2). We hypothesize that the higher rate of enrollment is due to increased levels of trust due to the pre-existing relationships between an individual and health care institution, given that 43% of 1754 adults in a national survey cited a lack of trust as a barrier to participation in clinical trials [33].

Minority recruitment was less than half of the goal (8.6% v. 18%), due to lower than expected rates of recruitment at Temple University, which has a large minority population yet a much lower number of patients to be recruited. In addition, rates of osteoporosis are lower among black women, further increasing the challenge of minority recruitment. In a systematic review, Ballane and colleagues observed that rates of vertebral fracture are 1.6 times higher in Whites than in Blacks [14]. One potential option to increase minority enrollment is to intensify recruitment of Hispanic populations, which have similar rates of osteoporosis as Whites [14]. This would need to be balanced, however, by observations that the costs of recruiting minority populations that often do not speak English are up to five times as high as recruiting non-minority populations [34].

Though the WISE study was designed according to pragmatic trial principles [11], where tradeoffs are made to limit individuals excluded, our screening enrollment rate was not as low as in the STRIDE study, also designed using pragmatic principles. The LIFE Study, designed as an efficacy study, screened 9.0 individuals for each person enrolled, 50% higher than in WISE where 6.0 individuals completed a telephone interview for each individual enrolled [31] but nearly three times higher than the STRIDE study, which only screened 3.4 individuals for each participant enrolled [32]. Two potential reasons may explain these differences. First, our recruitment letter did not focus attention on the need to have a fragility fracture in the past, which may partly explain why not having a fragility fracture was the reason for the vast majority (87%) of exclusions. Past history of fragility fracture also has no field in the electronic health record that may have made it easier to identify potential participants. For example, Fu and colleagues identified 10,898 smokers in an electronic health record, as smoking status is recorded at each visit, screening only 1.7 individuals for each participant enrolled [35]. Second, our intervention was significantly more demanding to participants than STRIDE. WISE required participants to consider participating in a 3-year intervention trial, including ~ 450 h of home or center-based intervention time (three times weekly × 150 weeks). The STRIDE study, by comparison, studied the impact of a falls care manager who provided additional clinical services (e.g., medication changes), but that would not require a large time and effort commitment [32, 36].

The population we recruited is representative of those who are at the highest risk of falls and fall-related injuries. While the STRIDE study is a primary prevention trial and WISE is a secondary prevention trial, the populations are quite similar. The WISE study had a higher percentage of women than the STRIDE study (84% versus 62%), likely due to the STRIDE study’s goal to enroll individuals at risk for falls, where the WISE study aimed to enroll individuals with a previous fragility fracture, a marker of osteoporosis. Approximately 80% of individuals with osteoporosis are female [37], similar to the demographics of the WISE study.

A key lesson from the recruitment phase of WISE is the importance of considering a composite clinical outcome, rather than an individual clinical outcome, as a means of simplifying recruitment challenges. We originally powered the study to detect a difference in fragility fracture, which required 2000 participants to detect a difference in rates of incident fragility fracture. We did, however, include detailed power analyses for a secondary outcome measure, a composite outcome measure of fragility fracture and serious fall-related injury (FF/SFRI). This composite outcome measure had a higher incidence, as it includes a wider range of events, and therefore requires fewer participants to detect a significant difference between conditions [38]. Composite outcomes are increasingly used in studies of other clinical conditions, including cardiovascular disease, by combining incident cardiovascular death or hospitalization for myocardial infarction or stroke [39]) and chronic obstructive pulmonary disease (COPD), by combining the incident need for intubation, death from any cause, hospital admission for COPD or intensification of drug therapy [40]. Our experience shows that other clinical conditions may also take advantage of composite outcomes when designing clinical trials.

Although this study has a number of strengths, it has limitations as well. First, although the WISE study was performed in three regions of Pennsylvania spanning a distance of 300 miles, recruitment results may have differed in other regions. Pennsylvania has one of the highest percentages of citizens over the age of 65, ranking 8th out of 50 states, with 18.2% of citizens aged 65 or older. California, for example, ranks 45th out of 50, with only 14.3% of citizens aged 65 or older. While this demographic difference may have led to a different level of responsiveness to mailings and other recruitment efforts, the multi-center Lifestyle Interventions and Independence for Elders observed that telephone screens yielded a similar percent of eligible participants across eight geographically diverse centers, ranging only from 61% to 79%, with an average of 68% [31]. Second, WISE study participants were more likely to have graduated college (54.9%) than older adults in the United States overall (38.0%) [41], potentially creating a threat to the generalizability of the findings. Despite these differences from the overall population, the WISE study recruited a similar percentage of college graduates as the STRIDE study (52.2%) and fewer than the LIFE Study (64.2%). These findings highlight the importance of improving outreach to recruit participants of lower educational attainment, so that the study sample more accurately reflects the population under study,

In conclusion, the WISE study enrollment period demonstrates the feasibility of recruiting a large population of individuals at risk for fall-related injuries primarily using mailed letters, which can be scaled up and down relatively easily to meet recruitment goals. Also, the data demonstrate that having multiple sites with different demographic characteristics increases the chances of recruiting a sample with diverse demographic and other characteristics that are covariates of the outcome of interest. Lastly, the recruitment experience highlights the need, where possible, to consider other clinically-relevant outcomes that can be combined into a composite outcome, in order to lower sample size requirements, given how frequently recruitment challenges arise in clinical trials.
