# Correction to: Working to increase stability through exercise (WISE): screening, recruitment, and baseline characteristics

**DOI:** 10.1186/s13063-022-06018-0

**Published:** 2022-01-14

**Authors:** Christopher N. Sciamanna, Noel H. Ballentine, Melissa Bopp, Vernon M. Chinchilli, Joseph T. Ciccolo, Gabrielle Delauter, Abigail Fisher, Edward J. Fox, Suzanne M. Jan De Beur, Kalen Kearcher, Jennifer L. Kraschnewski, Erik Lehman, Kathleen M. McTigue, Edward McAuley, Anuradha Paranjape, Sol Rodriguez-Colon, Liza S. Rovniak, Kayla Rutt, Joshua M. Smyth, Kerry J. Stewart, Heather L. Stuckey, Annie Tsay

**Affiliations:** 1 Department of Medicine, Division of General Internal Medicine, Penn State College of Medicine, Box HO34, 500 University Drive, Hershey, PA 17033 USA; 2 Penn State College of Medicine, Hershey, USA; 3 Penn State University, State College, USA; 4 Columbia University, New York City, USA; 5 Johns Hopkins University, Baltimore, USA; 6 University of Pittsburgh, Pittsburgh, USA; 7 University of Illinois, Urbana, USA; 8 Temple University, Philadelphia, USA

**Correction to: Trials 17:809 (2021).**

**https://doi.org/10.1186/s13063-021-05761-0**

Following the publication of the original article [1], we were notified of an error in last author’s name.

Originally published name: Anne Tsay.

Corrected name: Annie Tsay.

The original article has been corrected.

## References

[1] Sciamanna CN (2021). Working to Increase Stability through Exercise (WISE): screening, recruitment, and baseline characteristics. Trials.

