# Correction: Working to Increase Stability through Exercise (WISE): screening, recruitment, and baseline characteristics

**DOI:** 10.1186/s13063-026-09811-3

**Published:** 2026-05-28

**Authors:** Christopher N. Sciamanna, Noel H. Ballentine, Melissa Bopp, Vernon M. Chinchilli, Joseph T. Ciccolo, Gabrielle Delauter, Abigail Fisher, Edward J. Fox, Suzanne M. Jan De Beur, Kalen Kearcher, Jennifer L. Kraschnewski, Erik Lehman, Kathleen M. McTigue, Edward McAuley, Anuradha Paranjape, Sol Rodriguez-Colon, Liza S. Rovniak, Kayla Rutt, Joshua M. Smyth, Kerry J. Stewart, Heather L. Stuckey, Annie Tsay

**Affiliations:** 1 Department of Medicine, Division of General Internal Medicine, Penn State College of Medicine, Box HO34, 500 University Drive, Hershey, PA 17033 USA; 2 Penn State College of Medicine, Hershey, USA; 3 Penn State University, State College, USA; 4 Columbia University, New York City, USA; 5 Johns Hopkins University, Baltimore, USA; 6 University of Pittsburgh, Pittsburgh, USA; 7 University of Illinois, Urbana, USA; 8 Temple University, Philadelphia, USA

**Correction: Trials 22, 809 (2021)**

**https://doi.org/10.1186/s13063-021-05761-0**

Following the publication of the original article [1], we were notified that Tables 4 and 5 each need to have a footnote at the bottom to address missing data.

Under Table 4 the footnote should read: "Due to missing data, which differed for each variable, group sizes (N) ranged as follows: Overall (1038- 1139); Control (512-569); Intervention (526-570).

Under Table 5 the footnote should read: "Due to missing data, which differed for each variable, group sizes (N) ranged as follows: Overall (1038-1139); Penn State (443-501); Pitt (487-526); Temple (108-112)".

Proposed sample size was 2100, not 2000 as was noted on page 3. This change was made between the funding announcement and the beginning of recruitment, to provide additional power to detect proposed differences.

The original article has been corrected.
